# Humanized yeast to model human biology, disease and evolution

**DOI:** 10.1242/dmm.049309

**Published:** 2022-06-06

**Authors:** Aashiq H. Kachroo, Michelle Vandeloo, Brittany M. Greco, Mudabir Abdullah

**Affiliations:** Centre for Applied Synthetic Biology, Department of Biology, 7141 Sherbrooke St. W, Concordia University, Montreal, QC H4B 1R6, Canada

**Keywords:** Functional complementation, Functional replaceability, Humanized yeast, Orthology

## Abstract

For decades, budding yeast, a single-cellular eukaryote, has provided remarkable insights into human biology. Yeast and humans share several thousand genes despite morphological and cellular differences and over a billion years of separate evolution. These genes encode critical cellular processes, the failure of which in humans results in disease. Although recent developments in genome engineering of mammalian cells permit genetic assays in human cell lines, there is still a need to develop biological reagents to study human disease variants in a high-throughput manner. Many protein-coding human genes can successfully substitute for their yeast equivalents and sustain yeast growth, thus opening up doors for developing direct assays of human gene function in a tractable system referred to as ‘humanized yeast’. Humanized yeast permits the discovery of new human biology by measuring human protein activity in a simplified organismal context. This Review summarizes recent developments showing how humanized yeast can directly assay human gene function and explore variant effects at scale. Thus, by extending the ‘awesome power of yeast genetics’ to study human biology, humanizing yeast reinforces the high relevance of evolutionarily distant model organisms to explore human gene evolution, function and disease.

## Abstract (precis)

**Summary:** The functionally replaced human genes in yeast generate humanized yeast, providing a simple growth-based readout for the functions of hundreds of critical human genes and allowing researchers to study them in a simplified organismal context.

## Introduction

Conservation of the building blocks of life, ease of genetic manipulation, well-established procedures for propagation and testing in laboratory settings are the fundamental reasons scientists use model systems to understand biological processes (Alberts, 2010; Hedges, 2002). Despite their simplicity, the budding yeast *Saccharomyces cerevisiae* shares many essential cellular processes with humans, serving as a widely popular model for basic research. Several decades of research on budding and on the fission yeast *Schizosaccharomyces pombe* have contributed to understanding critical conserved cellular processes, enabling our understanding of human biology and diseases like cancer (Duina et al., 2014; Hoffman et al., 2015). From elucidating fundamental molecular and cellular mechanisms to serving as a proxy to study human disease analogs, yeast is an invaluable tool. Its impact is reflected in the five Nobel prizes awarded for discoveries in yeast: cell cycle (2001), transcription (2006), telomeres (2009), vesicle transport (2013) and autophagy (2016) (Hohmann, 2016). Furthermore, given the availability of several genome-wide knockout, temperature-sensitive and regulatable promoter collections, yeast has emerged as a model eukaryote for functional genomic screens (Li et al., 2011; Pan et al., 2004; Winzeler et al., 1999; Boone, 2014; Botstein and Fink, 2011; Scherens and Goffeau, 2004). Combined with excellent genetic tractability, easy conversion between haploid and diploid forms and superabundance of genetic tools, *S. cerevisiae* is arguably the pioneer model for eukaryotic synthetic and systems biology research.

Yeast is the first eukaryote to have its genome sequenced and synthetically designed (Richardson et al., 2017;  Goffeau et al., 1997). In particular, the yeast genetic interactions (GIs) and protein–protein interactions (PPIs) have been mapped extensively (VanderSluis et al., 2018). GIs represent functional interactions between genes or their products that often directly impact genotype–phenotype relationships and diverse aspects of biology from evolution to disease (Phillips, 2008). GIs can be quantified when the combination of two (bi-allelic) or >2 (tri-allelic or more) mutations in distinct genes yield a lethal (synthetic lethal), sub-lethal (synthetic negative) or hyperactive (synthetic positive) phenotype (van Leeuwen et al., 2017). Over the past two decades, the discovery of bi- and tri-gene–phenotype associations have spiked to millions, covering nearly the entire yeast genome [available at Saccharomyces Genome Database (SGD) (Cherry, 2015; Costanzo et al., 2016; Kuzmin et al., 2018)]. This near-complete global GI map makes yeast an ideal model to link genotypes to phenotypes while providing a reference to guide the discovery of human GIs.

Model organism databases provide immediate access to vital information and significantly contribute to our understanding of human biology and disease (Bellen et al., 2021; Leonelli and Ankeny, 2012). Additionally, fully annotated gene and protein sequences, and orthology and ontology databases deliver the impetus to further characterize the fundamental and translational concepts in human biology (Alliance of Genome Resources Consortium, 2019; Alliance of Genome Resources Consortium, 2020; Leonelli and Ankeny, 2012; UniProt Consortium, 2021). Despite their many advantages, model organisms are not exact copies of humans, and researchers need to exercise caution when extrapolating results. Successful translation thus requires a new direction in model organism research with direct human applications (Rine, 2014). By leveraging our deep understanding of yeast biology and genetic resources, this Review outlines novel strategies for high-throughput systems genetics and synthetic biology to study human biology, disease and evolution directly in a simplified organismal context.

## Using model organisms to discover gene–phenotype associations in humans

A significant challenge in human biology is attributing heritable changes in the genome to a particular phenotype, except in the case of Mendelian diseases. The difficulty is partly due to insufficient systematic information on higher-order genetic interactions, underpowered sample sizes and the lack of knowledge of pedigree. Other factors contributing to the complexity of genome–phenotype associations are pleiotropy and context-specific phenotypic variations, which geneticists call ‘epistasis’ (Young et al., 2019). Although tools such as genome-wide association studies (GWAS) enable the discovery of complex genetic associations in disease, solving this problem using model systems would further validate the disease-causing genes. In addition, while tools such as CRISPR/Cas9 have opened up avenues to systematically discover GIs in human cells (Costanzo et al., 2019), this endeavor will require several more years of work. Until then, as has often been the case before, the solution could lie in evolutionary conservation.

The underlying principle is simple: all organisms are descendants of a common ancestor; thus, all organisms share genes. This is the unifying reasoning behind using model organisms in biomedical research (Hunter, 2008). However, this line of research has often puzzled biologists; for instance, when mutated conserved genes result in different phenotypes among different organisms (Liao and Zhang, 2008; McGary et al., 2010). The problem becomes even more challenging in highly diverged model organisms such as yeast or bacteria. How does one correlate conserved function between distantly related organisms and humans if the phenotypes resulting from altered gene functions are so different? For example, cell cycle genes in yeast provide a direct insight and link to cancer in humans (Hartwell, 2004; Nurse, 2002). Conversely, yeast genes involved in cell wall biosynthesis, although conserved, yield very little information about their function in blood vessel formation in humans (Hillenmeyer et al., 2008; McGary et al., 2010). This apparent conundrum does not imply diverged function. On the contrary, despite being conserved, genes and their interactions can be repurposed to generate diverse phenotypes in different species (Dryja et al., 1984; Lu and Horvitz, 1998, 53; Roscito et al., 2018).

Categorizing conserved genes among species based on their mutant phenotypes could, therefore, inform gene–phenotype associations in diverged organisms (McGary et al., 2010). Two distinct phenotypes in distant species showing a significant overlap of conserved genes are referred to as orthologous phenotypes or simply ‘phenologs’ (McGary et al., 2010). Therefore, using the enormous amount of data already generated in yeast and other model organisms could, in part, compensate for the lack of meaningful genotype–phenotype data in humans. McGary et al. (2010) found many such associations, discovering equivalent gene modules involved in human diseases and mutant phenotypes in model organisms (http://www.phenologs.org/). Additionally, better predictions should emerge as more GI data from model organisms, particularly higher eukaryotes, are obtained (Golden, 2017; McWhite et al., 2015; Woods et al., 2013).

However, although linking diverged phenotypes to conserved genes is fascinating, it assumes that the shared genes separated by millions of years of evolution have maintained identical or similar functions (Botstein and Fink, 1988; Gabaldón and Koonin, 2013). Therefore, estimating functionally equivalent genes among species is a vital endeavor. *S. cerevisiae* provides an advantageous platform to systematically test the functional ‘replaceability’ of shared genes, thus extending the notion of functional similarity beyond sequence conservation and phylogeny. Throughout this Review article, we use the term ‘functional replaceability’ or ‘humanization’ to define the ability of a human gene to complement (replace) its yeast counterpart.

## Why humanize yeast?

**Fig. 1.**
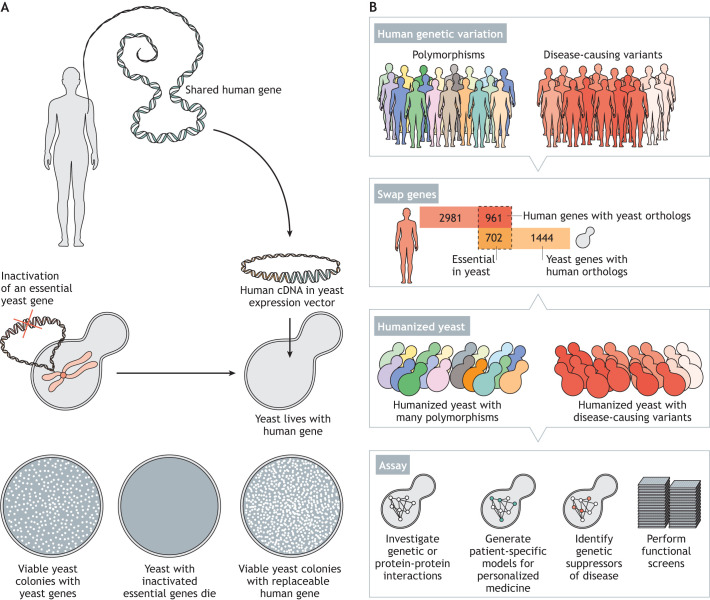
**Swapping conserved human genes in yeast.** (A) General outline of yeast humanization assays. Shared human protein-coding sequences are cloned in yeast expression vectors. The wild-type yeast that express functional essential genes are viable, as indicated by colonies growing on Petri dishes. The deletion or conditional knockout of the essential yeast gene causes lethality, resulting in an absence of colonies on Petri dishes. However, yeast are viable if the expression of the orthologous human gene can compensate for the function of the yeast counterpart, despite nearly a billion years of divergent evolution, as indicated by growth on Petri dishes similar to that of wild-type yeast. (B) There is extensive genetic polymorphism in critical human genes, and some of these mutations often lead to disease. Yeast shares 2146 orthologs with humans, of which 702 are essential in yeast. Comparatively, owing to gene amplification, humans share 3942 genes with yeast, of which 961 human orthologs relate to corresponding essential counterparts in yeast [data obtained from Inparanoid (Sonnhammer and Östlund, 2015)]. By functionally replacing the conserved human genes in yeast, the resulting humanized yeast become a tractable system for testing human genetic variation in the context of a simplified cell. These yeast–human gene swaps allow researchers to characterize genetic or protein–protein interactions relevant to disease, build entire human pathways in yeast, generate personalized yeast strains for each unique human variant, identify genetic suppressors of human disease, and provide a platform to identify novel therapeutics. Image concept credit: Andrew Horton.

The remarkable extent to which genes are functionally conserved between humans and yeast demonstrates the power of distant model organisms for studying human processes (Dixon et al., 2008; Dolinski and Botstein, 2007; Sonnhammer and Östlund, 2015; Tugendreich et al., 1994). In particular, budding yeast shares >2000 genes (∼30% of its genome) with humans (Fig. 1). Furthermore, yeast's genomic DNA is easy to manipulate with modern genetic tools such as CRISPR/Cas9 (Akhmetov et al., 2018; DiCarlo et al., 2013; Lee et al., 2015). Yeast has innately high homologous recombination rates that simplify genomic manipulation. Additionally, yeast can be transformed with DNA molecules that are maintained within a cell as single-copy (centromere origin) or multicopy (2-micron origin) plasmids or as artificial chromosomes (Aylon and Kupiec, 2004; Chan et al., 2013; Kumar and Snyder, 2001; Shashikant et al., 1998; Tschumper and Carbon, 1983).

The rationale for using budding yeast for systematic humanization assays has primarily relied on the availability of genome-wide knockout, temperature-sensitive and titratable promoter yeast collections, and the human ORFeome collection (Lamesch et al., 2007) comprising human protein-coding complementary DNAs (cDNAs) that are compatible with yeast expression vectors for high-throughput cloning (Giaever et al., 2002; Li et al., 2011; MGC Project Team et al., 2009; Mnaimneh et al., 2004; Pan et al., 2004; Winzeler et al., 1999; Yang et al., 2011). Furthermore, given their rapid growth in laboratory conditions, simple conversion between easily discernible haploid and diploid forms, availability of many selection markers and genome-wide mutant libraries, yeast is a unique model for large-scale testing of orthologous human gene function. Finally, the humanization of yeast is efficient and straightforward. A typical humanization assay involves cloning of the orthologous human cDNA in yeast expression vectors, followed by transformation in yeast strains harboring conditional knockout alleles of the corresponding yeast genes (Kachroo et al., 2015). If the yeast gene is essential, viable yeast cells that express the human gene alone suggest functional replaceability (Fig. 1A). Alternatively, CRISPR/Cas9 enables precise and markerless insertion of human coding sequences at the corresponding genomic loci, thus replacing the yeast gene while maintaining the native regulation (Akhmetov et al., 2018; Brzácˇová et al., 2021). Therefore, humanization assays provide a simple growth-based readout of function while bringing together all of the benefits of using yeast cells to study human genetics (Botstein and Fink, 2011; Laurent et al., 2016).

In spite of the cellular and organismal differences between humans and yeast, several recent studies have discovered that many conserved protein-coding human genes can substitute for their yeast equivalents and sustain yeast growth (Garge et al., 2020; Hamza et al., 2015, 2020; Kachroo et al., 2015; Laurent et al., 2020; Sun et al., 2016). These assays uncovered which conserved processes are still interchangeable while also creating reagents to directly test human gene function in a tractable system. Thus, humanized yeast serve as biological reagents in a simplified organismal context, opening up simple high-throughput assays of human gene function, measuring the impact of human genetic variation on function, the screening and repurposing of drugs, and the rapid determination of mechanisms of drug resistance and specificity (Fig. 1B).

Furthermore, humanized yeast enable testing of the effects of human genetic variation on function at scale. Each strain carrying a unique variant of a human gene provides a synthetic platform to decipher the impact of mutations without confounding parameters such as gene expression, GI or physical interaction context in humans. Indeed, several studies have used yeast to assay for human gene function, such as characterizing human p53 mutations (Ishioka et al., 1993; Smardová, 1999) and modeling Huntington’s disease to identify human kynurenine 3-monooxygenase as a therapeutic target (Giorgini et al., 2005). Furthermore, a recent study used humanized yeast to discover the human target of the U.S. Food and Drug Administration (FDA)-approved antifungal drug thiabendazole, potentially repurposing the drug to inhibit neoangiogenesis, a hallmark of cancer (Garge et al., 2021). This and other examples (Bach et al., 2003; Friedmann et al., 2006; Perkins et al., 2001) strongly support using humanized yeast as a platform to characterize human disease mutations and develop novel therapeutics (Fig. 1).

## Humanized yeast for modeling human genetic variation and disease

Large-scale human genome sequencing data have uncovered extensive genetic polymorphism in our genes, including many rare variants that cause or predispose to diseases (1000 Genomes Project Consortium et al., 2015; Lek et al., 2016). However, studying how this variation contributes to cellular function and overall human health remains a challenge, particularly as the rate of discovery of variants of unknown significance (VUS) has increased dramatically (Koboldt et al., 2013). Humanized yeast offer a possible solution.

**Fig. 2.**
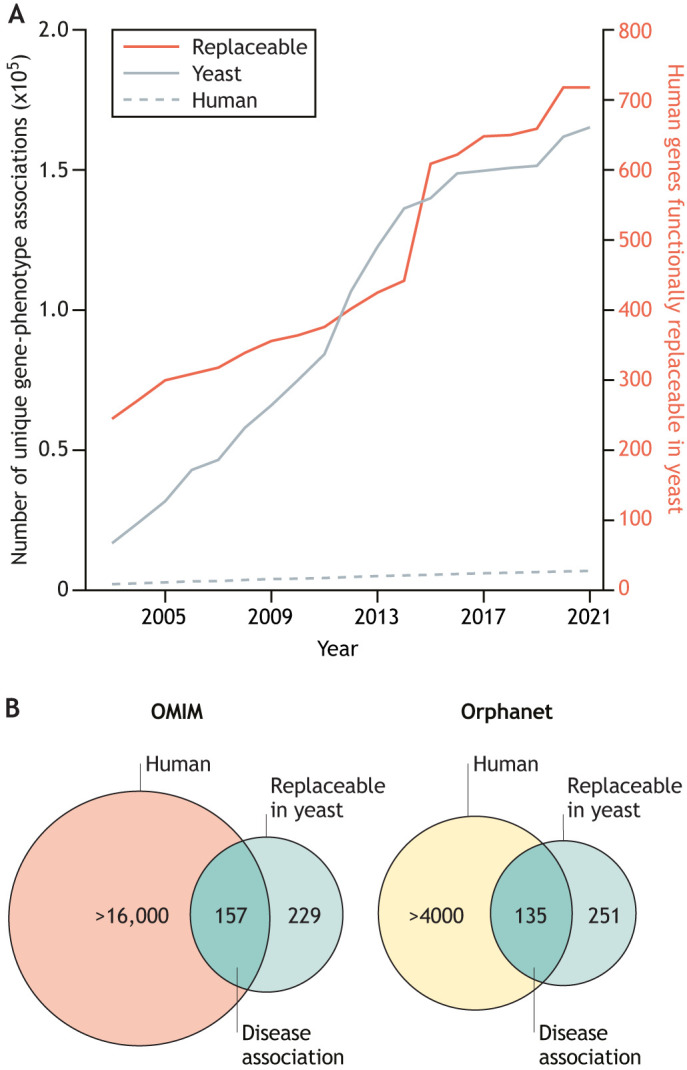
**Humanized yeast is an advantageous system to model human disease.** (A) The past two decades of research have revealed millions of gene–phenotype associations in yeast. The number of known single gene–phenotype associations in yeast (gray solid; left *y*-axis) has surpassed those known in humans (gray dashed; left *y*-axis). Similarly, many human genes are functionally replaceable in yeast (red; right *y*-axis) [data obtained from SGD, YeastMine (Balakrishnan et al., 2012)]. (B) Humanizable essential genes in yeast overlaid with genes from the Online Mendelian Inheritance in Man (OMIM) database. Of the 386 essential yeast genes that can be functionally replaced with human orthologs, 157 have disease association in the OMIM database. Similarly, of the 386 functionally replaceable essential yeast genes, 135 overlap with rare disease-associated genes in the Orphanet database [data obtained from OMIM and Orphanet (Amberger et al., 2015; Weinreich et al., 2008)]. See Table S2 for complete gene lists.

Many years of research have dissected every aspect of yeast biology. As a result, we know a lot about yeast genes and their associated phenotypes. Compared to yeast, human gene–phenotype associations have had a much slower discovery rate, with the data exclusively contributed to Mendelian disease association via the Online Mendelian Inheritance in Man (OMIM) database (Fig. 2A) (Amberger et al., 2015). Similarly, over the past few decades, the number of functionally replaceable human genes in yeast has also increased to >700 (Fig. 2A; Table S1) (Balakrishnan et al., 2012). Notably, many replaceable human genes encoding critical biological processes have overlapping Mendelian and rare disease associations (Fig. 2B; Table S2), making the humanized yeast carrying these genes a unique model of human diseases.

The principle behind the approach entails replacing the yeast genes with their disease-associated human counterparts, linking the fitness of a variant human protein with the fitness of the yeast cell, particularly if the gene is essential for optimal growth and survival (Fig. 1). Thus, missense or nonsense mutations with functional consequences should show phenotypic differences in the humanized yeast, such as a slower growth rate. Therefore, humanized yeast growth fitness serves as an easily measured proxy for the proper functioning of the human gene (Kachroo et al., 2015; Marini et al., 2008; Sirr et al., 2020; Sun et al., 2016). The success of this strategy relies on the awesome power of yeast genetics and on a plethora of readily available tools that allow researchers to constitutively express or modulate the expression of human genes in yeast.

**Fig. 3.**
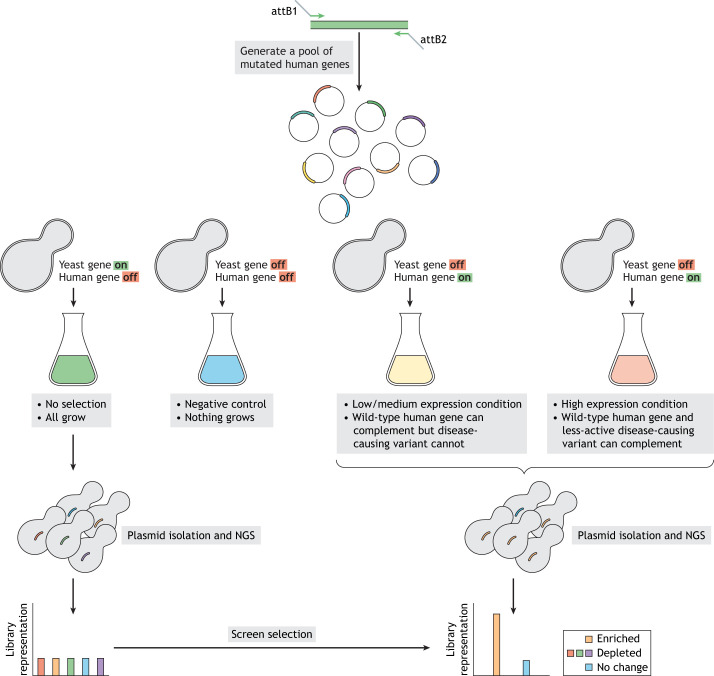
**Deciphering the impact of human genetic variation in yeast.** Experimental pipeline for high-throughput screening to interpret human genetic variation in yeast. A library of mutant human genes is generated by either error-prone PCR or by using a mutated oligonucleotide pool, cloned in yeast expression vectors and transformed into a suitable yeast strain in which the orthologous yeast gene can be inactivated by conditional repression or knocked out. The transformed yeast cells are grown under various conditions that control which allele is expressed, either the endogenous yeast one only (green culture) or the human one only – at low/medium expression levels (yellow culture), high expression levels (orange culture) or neither (blue culture). These four gene expression conditions will have different effects on the growth of individual yeast cells harboring unique human gene variants. The culture that expresses yeast alleles only serves as the ‘baseline’ readout of the overall variant human pool. The human gene expression conditions select for human gene variants based on whether their activity translates into their ability to sustain the growth of the yeast cells. Yeast cells that express deleterious human gene variants are eliminated from the pool. After selection, the plasmid pool from each growth condition is sequenced. Computational analysis of reads corresponding to each variant in the pool quantifies the fitness of each variant. The frequency of a particular variant relative to the baseline readout identifies deleterious mutations likely associated with human disease. NGS, next-generation DNA sequencing.

Moreover, with the improvement of efficient and less-costly next-generation DNA sequencing (NGS) technologies and deep mutational scanning (DMS) strategies (Fowler et al., 2010, 2014; Starita and Fields, 2015), humanized yeast opens up avenues to address the impact of every amino acid change in a human protein on its function, providing a functional readout of each variant, even those currently designated as VUS or the as yet unidentified variations (Kuang et al., 2020; Sun et al., 2020; Weile et al., 2017). In a typical DMS workflow, a pooled library of human mutant genes is constructed by error-prone PCR or by synthesizing mutant oligonucleotides (Weile et al., 2017). The library is cloned in single-copy plasmids and introduced into equivalent conditional-knockout yeast strains. Next, the pooled yeast strains are grown in restrictive conditions to inactivate or segregate away the yeast gene and select the human variants that support cell growth in the absence of yeast gene function. Finally, the plasmids from the growing yeast strains are isolated, and the variants are identified by NGS. A similarly prepared culture of the starting library grown in unselective conditions serves as the baseline for estimating the fitness defects, as any decreased sequence representation in the competitive growth experiment is assessed relative to variant representation in this baseline culture. Therefore, neutral variants can be easily distinguished from deleterious ones (Sun et al., 2020; Weile et al., 2017). Thus, humanized yeast growth fitness serves as an easily measurable and reasonably nuanced proxy for assessing the functioning of a human gene (Fig. 3). However, the strategy is restricted to functionally replaceable human genes in yeast.

Although high-throughput DMS assays provide a functional map of human gene function at a single-amino acid resolution, the assays only reveal deleterious or low-activity variants and do not identify hyperactive mutants associated with human disease. For example, hyperactive variants of human cystathionine beta-synthase were classified as wild type in a pooled yeast DMS assay (Weile et al., 2017). Implementing titratable promoters may distinguish hyperactive variants, and subsequent biochemical characterization like testing for the catalytic activity, stability and folding kinetics may identify the contributing features of specific hyperactivity-conveying amino acid changes (Fig. 3). Furthermore, the growth-based assays do not provide a mechanistic understanding of the loss-of-function phenotypes of human genes. Gaining deeper insights requires characterizing each variant biochemically or inferring mechanism using structural data, which is now possible for any protein with the artificial intelligence-based AlphaFold (Jumper et al., 2021). However, despite advances in the acquisition of protein structure data, evidence for variant classification utilizing the structure and computational tools alone has been weak at best, and, in many instances, yeast complementation assays, like DMS, capture more pathogenic variants than computational prediction methods (Marini et al., 2008; Sun et al., 2020). Given that the American College of Medical Genetics and Genomics and the Association for Molecular Pathology guidelines consider experimental assays of variant function to be among the strongest sources of evidence for variant classification, yeast-based complementation assays are a powerful tool to assay for human genetic variation at scale. Additionally, DMS-based assays can exploit the growing number of replaceable paralogous human–yeast gene relationships for identifying disease variants, with the performance of these assays on par with that of orthology-based assays (Laurent et al., 2020; Yang et al., 2017).

## Gene swaps reveal principles governing functional conservation

**Fig. 4.**
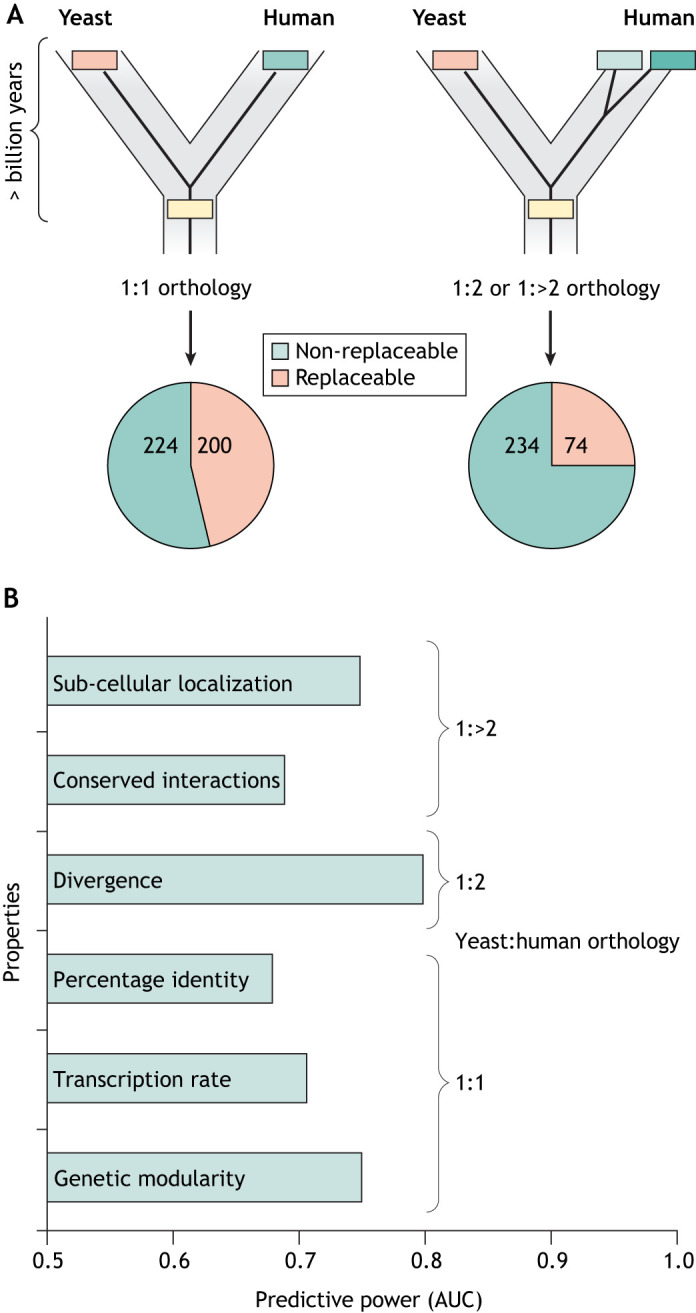
**Systematic humanization of yeast reveals the properties critical for functional replaceability.** (A) Yeast and human genomes share several thousand orthologs that belong to different classes. 1:1 orthologs are shared genes that have acquired no observable duplications in either lineage, whereas 1:2 or 1:>2 refers to orthologs that have undergone duplication in humans (Laurent et al., 2020). Our previous work shows that ∼40% of the tested human genes can functionally replace their yeast counterparts comprising 1:1, 1:2 or 1:>2 orthologs (Kachroo et al., 2015; Laurent et al., 2020). (B) Large-scale replaceability assays identify critical features of shared genes important for functional complementation in yeast. In 1:1 orthologs, genetic modularity is the best predictor of replaceability, followed by transcription rate and amino acid sequence identity. By contrast, in 1:2 or 1:>2 orthologs, the top predictors are divergence of the human or yeast genes, conserved interactions and similar sub-cellular localization. The *x*-axis represents the predictive power calculated as area under the curve (AUC) or receiver operator curve plots [computed from data in Kachroo et al. (2015) and Laurent et al. (2020)].

Functional complementation tests of human genes in yeast are not novel and have been explored extensively in the past (Balakrishnan et al., 2012; Heinicke et al., 2007). However, a systems approach to test hundreds of shared human genes for functional replaceability in yeast has only recently been reported (Garge et al., 2020; Hamza et al., 2015, 2020; Kachroo et al., 2015; Laurent et al., 2020; Sun et al., 2016) (Fig. 4A). The large-scale nature of the studies enabled the investigation of hundreds of properties of shared genes, revealing features, some rather unexpected, governing functional replaceability (Hamza et al., 2015; Kachroo et al., 2015, 2017; Laurent et al., 2020). For example, the data showed that sequence identity or similarity is not the best predictor of functional replaceability (Fig. 4B) (Kachroo et al., 2015). This research is crucial, as biologists have thus far heavily relied on indirect properties such as sequence conservation as the predictor of functional replaceability, even though data show that attempts to predict complementation based only on sequence similarity between human and yeast genes have not been successful (Marini et al., 2010).

**Fig. 5.**
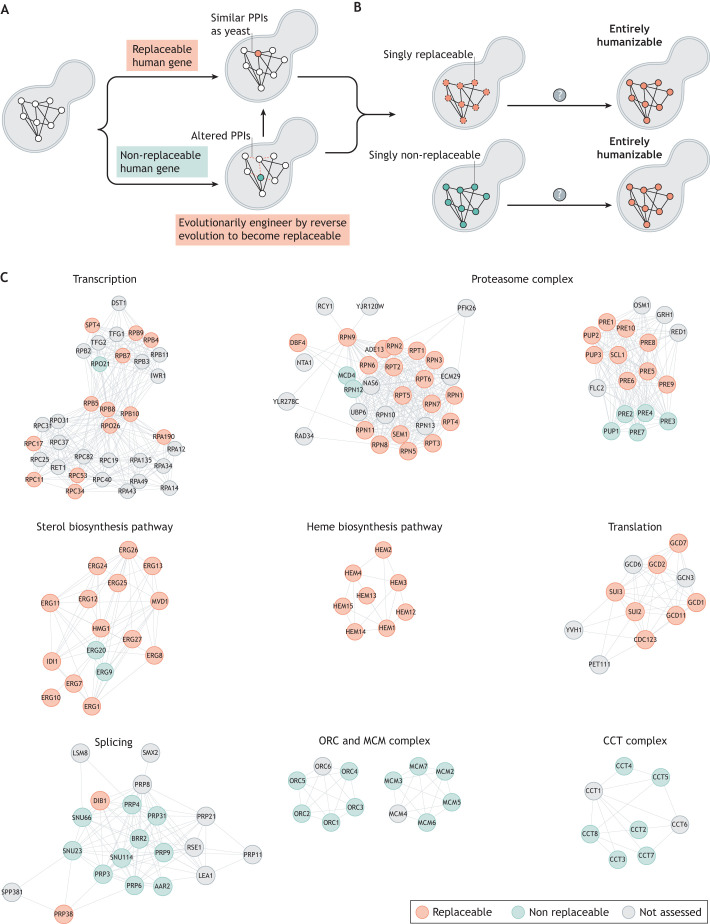
**Genetic modules govern functional replaceability.** (A) Lack of functional complementation by a shared human gene in yeast could be attributed to the inability of a human gene to perform critical genetic interactions or protein–protein interactions (PPIs) in yeast. Using a reverse evolution approach and modifying a non-replaceable human gene to complement the yeast ortholog should allow the discovery of critical interactions or other factors, such as diverged mechanisms or regulation between humans and yeast. (B) Genetic modularity is a feature that strongly predicts replaceability and allows researchers to test whether higher-order humanizations of yeast are possible. Some modules can be humanized because most of the individual genes within the module are replaceable, either sequentially or by expressing all humanized components simultaneously. However, non-replaceable modules represent a major challenge and could be humanized if the entire yeast genetic module is replaced simultaneously [as in Truong and Boeke (2017)]. (C) Several yeast genes are functionally replaceable by their human equivalents one gene at a time, but many are not. For example, genes encoding components of the transcription and translation machinery, the proteasome complex, and the sterol and heme biosynthesis pathways are mostly replaceable. By contrast, modules such as the splicing complex, the origin recognition complex (ORC), minichromosome maintenance (MCM) complex and the chaperone-containing TCP-1 (CCT) complex are largely non-replaceable. This distribution of replaceable or non-replaceable human genes in pathways or complexes suggests that these yeast processes are likely humanizable in their entirety, even when individual genes are non-replaceable. The module maps were generated using Cytoscape (version 3.9.1) (Shannon et al., 2003) with data from Garge et al. (2020), Kachroo et al. (2015) and Laurent et al. (2020), and are meant to illustrate the broad spectrum of functional replaceability across different cellular processes.

In human–yeast orthologous pairs that have undergone no observable duplication in either lineage (referred to as 1:1 orthologs; Fig. 4A), the replaceability was mostly determined at the level of pathways and protein complexes rather than at the level of individual genes. This property, called genetic modularity, broadly defines the human protein's ability to interact with yeast proteins in a manner similar to that of the replaced yeast protein (Fig. 4B). Data showed that some genes that form a functional genetic module were entirely replaceable, such as those encoding components of the proteasome complex and of the sterol and heme biosynthesis pathways. By contrast, other modules, such as the DNA replication initiation complex, were altogether non-replaceable (Kachroo et al., 2015) (Fig. 5C). The same property was highly predictive in a systematic ‘bacterialization’ of yeast with 1:1 *Escherichia coli* orthologs, suggesting that genetic modularity could be a broadly predictive feature of replaceability in diverse species (Kachroo et al., 2017). These assays revealed that local physical (protein complexes), biochemical (metabolic pathways) and genetic interactions were highly predictive of gene replaceability.

For human–yeast orthologous pairs that have undergone lineage-specific duplication in the human lineage, referred to as 1:2 or 1:>2 orthologs (Fig. 4A), the systematic replaceability screens allow researchers to distinguish the shared genes that have either retained or lost ancestral functions after duplication (Laurent et al., 2020). This study also identified several features of shared genes predictive of functional replaceability. These include the relative divergence of the human co-orthologs from each other and from the yeast gene, where the least-diverged of the human co-orthologs is likely replaceable in yeast, involvement in GI or PPI networks, where the human co-ortholog that has retained more orthologous interactions is likely replaceable, and subcellular localization, where the human ortholog that localizes similarly to its yeast ortholog is likely replaceable (Fig. 4B) (Laurent et al., 2020).

## The curious case of non-humanizable yeast genes

The ortholog–function conjecture postulates the broad equivalence of functions among orthologs from diverse organisms (Gabaldón and Koonin, 2013). However, even if orthologous genes perform similar functions, it may not be possible to swap them if the organisms have widely diverged. For example, over half of the human genes tested by our own group are functionally non-replaceable in yeast (Kachroo et al., 2015; Laurent et al., 2020) (Fig. 5A). The absence of functional replaceability does not necessarily imply divergence of function, as replaceability is inherently a strict test for functional conservation, especially when using yeast cell growth as the only readout. However, functional replaceability indirectly measures the human gene's ability to perform most of the essential roles of the yeast counterpart, including critical GIs or PPIs (Zhong et al., 2016). Thus, restoring essential interactions over a certain threshold may enable the humanization of non-replaceable yeast genes. This hypothesis, of course, remains to be validated (Fig. 5A). However, results from the humanization of core histones and from a suppressor screen to identify replaceable human β2 proteasome subunit components in yeast suggest the lack of critical PPIs as a likely mechanism contributing to non-replaceability (Kachroo et al., 2015; Truong and Boeke, 2017).

Several factors can explain why conserved human genes are not replaceable in yeast. First, a yeast gene may need to evolve, accommodating the divergence of its partners to maintain efficient interaction over several million years of separate evolution, thus rendering the corresponding human ortholog incompatible (Fig. 5A) (Kachroo et al., 2015; Teufel et al., 2019). Second, recent data on most conserved human and yeast genes, such as those encoding cytoskeletal components, suggest that distinct human GIs or PPIs contribute to non-replaceability in yeast (Garge et al., 2020). Third, non-native expression can lead to non-replaceability. For example, expression of human *UROS*, a gene involved in heme biosynthesis, from a heterologous yeast promoter is toxic to yeast. However, maintaining native yeast regulation of the same gene allows functional replaceability (Kachroo et al., 2017). Finally, the inability to functionally complement could be due to diverged function, although this is unlikely for most proteins in deeply conserved complexes and pathways.

A classical suppressor screen of non-replaceable human genes in yeast can isolate mutant replaceable human genes. For such a screen, an error-prone PCR strategy can generate a pool of variants to obtain mutants that can now support yeast growth, i.e. sequences with only a few amino acid changes, to enable the human gene variant to functionally replace the yeast counterpart. Using this approach, our group showed that the non-replaceable human *PSMB7*, encoding a β2 catalytic subunit of the human 20S proteasome core, needs only a single amino acid change to pack into the yeast proteasome core (Kachroo et al., 2015; Tomko and Hochstrasser, 2013). Several other non-replaceable human genes may similarly become replaceable in yeast if mutated. Therefore, finding and characterizing mutations in human or yeast genes that allow the assimilation of human genes or their products in yeast would be essential for discerning changes in human GIs/PPIs or regulatory pathways and for potentially uncovering novel mechanistic insights into human genetic systems.

Furthermore, most humanization assays have only tested the functional replaceability of individual genes. However, genes do not work in isolation. The modularity paradigm suggests investigating whether entire yeast and human systems are interchangeable. It also raises the hypothesis that an entirely humanized genetic module might replace the corresponding yeast system even when individual components are not humanizable, owing to specific PPIs that fail in the hybrid yeast/human system (Fig. 5B,C). Thus, although a high-risk strategy, this innovative genetic modularity-based approach could enable the humanization of the yeast biological processes in their entirety. For example, genetic modules such as the proteasome complex and the cholesterol and heme biosynthesis pathways can be fully humanized, because these modules are primarily composed of genes that are individually replaceable in yeast (Kachroo et al., 2015, 2017) (Fig. 5C). The success of previous similar attempts, such as the humanization of core histones and purine biosynthesis pathway in yeast (Agmon et al., 2020; Truong and Boeke, 2017), suggests that this endeavor is not impossible. The resulting yeast strains carrying multiple human genes encoding components of the same biological complex or process will be more suitable for screening human disease-associated alleles, because they maintain human-like local GIs or PPIs. Furthermore, these fully humanized yeast provide a unique opportunity to study polygenic diseases associated with variants belonging to the same protein complex or biosynthetic pathway, such as studying complex diseases like porokeratosis in mostly humanizable sterol biosynthesis pathway (Zhang et al., 2015).

## Expanding the scope of studying human genes in yeast

Functional replaceability of human genes in yeast provides a tractable system to test the fitness of human gene variants. Despite several systematic humanization screens, so far, only 718 human genes have been confirmed to be functionally replaced in yeast, while over 4500 human genes have Mendelian disease associations (Amberger et al., 2015; Balakrishnan et al., 2012). The yeast humanization strategy fails to address many disease-associated non-replaceable and non-orthologous human genes. Thus, it is imperative to look beyond orthology and functional replaceability assays or even beyond using a single model organism (budding yeast) to broaden the scope of humanization to other genetically tractable model systems.

**Fig. 6.**
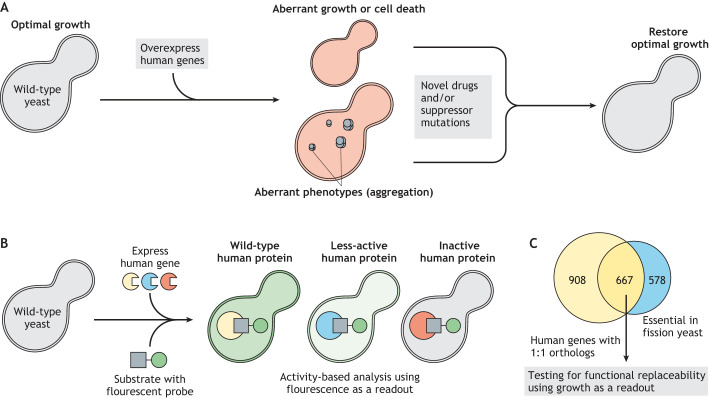
**Expanding the scope of humanized yeast.** (A) Systematic screening of human gene expression- or activity-mediated toxicity: these screens can identify novel therapeutics and genetic modifiers that restore optimal growth or phenotypes. In such screens, (over)expression of many human genes (of ∼15,000 genes in the human ORFeome collection) is typically toxic, resulting in a lethal yeast phenotype. However, applying chemical compounds or mutating the interacting genetic modifiers that abrogate the human gene's function restores yeast cell viability, allowing the discovery of potential therapeutics and likely genetic modifiers of disease. (B) Novel chemistry to assay human gene activity: measurable readouts, such as fluorescence, could identify disease-causing human gene variants in yeast. The strategy assays human gene function in yeast irrespective of orthology and essentiality. In Click-Seq, an example of such a strategy, the fully functional (wild-type) human gene product efficiently cleaves the substrate, resulting in intense green fluorescence of the probe. Sub-optimal cleavage activity of the mutated human gene product results in less-intense fluorescence, and the expression of a non-functional human gene results in an inactive protein that is unable to cleave the substrate and thus does not generate a fluorescent signal. (C) Functional replaceability testing: simply using growth as a readout, 667 human genes with clear, identifiable 1:1 fission yeast orthologs (essential) can be tested for functional replaceability [data obtained from InParanoid (Sonnhammer and Östlund, 2015) and Kim et al. (2010)].

Instead of aiming to replace an entire yeast gene, the classical approach of introducing human disease-associated mutations in endogenous yeast homologs has been widely successful (Aiyar et al., 2014; Couplan et al., 2011; Kucharczyk et al., 2009; Laurent et al., 2016; Rak et al., 2007). The strategy permits analyzing human genetic variation; however, the method requires the critical amino acids to be identical in the yeast homolog, limiting its use for many diverged human genes. Alternatively, human gene overexpression phenotypes, such as toxicity, can facilitate the functional characterization of many more human genes in yeast. Several systematic studies have identified human genes that are toxic when overexpressed in a wild-type yeast cell (Jo et al., 2017; Kachroo et al., 2015). Furthermore, a genome-wide approach testing several thousand human cDNAs identified >500 human genes that show expression-mediated or activity-mediated toxicity in yeast (Fig. 6A) (Sekigawa et al., 2010). This strategy is similar to the classical genetic approach of over/misexpression of the wild-type gene product (Prelich, 2012) and provides a simple yet powerful selection for human gene function with the potential to provide novel therapeutics. For example, overexpression of the wild-type human MAPK14 in yeast resulted in toxicity. However, the lethality was associated with aberrant activity rather than overexpression, as mutations that abrogate MAPK14 activity did not result in a toxic phenotype (Sekigawa et al., 2010). Friedmann and colleagues used MAPK14-mediated toxicity to yeast as a basis of a drug screen for compounds that can selectively inhibit MAPK14 activity. They identified a novel drug that, when applied to yeast, bypassed toxicity and thus effectively inhibited human MAPK14 (Friedmann et al., 2006). Similarly, other examples of human gene-mediated yeast toxicity include overexpression of human α-synuclein, TDP-43 (TARDBP) or human polyQ repeats (Bolognesi et al., 2019; Giorgini et al., 2005; Outeiro and Lindquist, 2003), supporting the use of this strategy to characterize human gene function in yeast.

Another promising advancement involves novel chemistry to measure human gene function. This particular process uses yeast as a surrogate to distinguish human gene variants based on the activity of their respective protein products. One such example, developed by Amorosi and colleagues (2021), is Click-Seq. Click-Seq uses enzymatic activity as a readout for massively parallel characterization of human gene variants in yeast. A substrate for a human enzyme is linked to a fluorophore. Upon cleavage, the released fluorophore emits fluorescence. Fluorescence-activated cell sorting separates single yeast cells based on the fluorescence readout (Fig. 6B), and the human gene-containing plasmids from the sorted cells are sequenced to identify variants that retain enzymatic activity. NGS of plasmids from non-fluorescing yeast cells reveals catalytically inactive human variants. A yeast Click-Seq screen for human *CYP2C9*, a highly polymorphic gene encoding a cytochrome P450 monooxygenase that metabolizes many molecules, including pharmaceuticals, identified variants that aberrantly metabolize drugs and are thus responsible for adverse drug effects (Amorosi et al., 2021) (Fig. 6B). In principle, similar methods could be employed for several other human genes, provided a readable output of their functions is available in a yeast cell.

Finally, fission yeast (*S. pombe*), a distant relative of budding yeast, provide a promising platform to complement the replaceability screening work in budding yeast. Because fission and budding yeast diverged over 400 million years ago, their spectra of replaceable genes may be different. Besides, the genome-wide knockout collection of fission yeast exists (Kim et al., 2010), and high-throughput expression vectors compatible with the human ORFeome collection are either already available or could be generated (Ahn et al., 2009). Data obtained from the InParanoid database reveal that nearly 4000 human genes have orthologs in fission yeast, ∼1200 of which are well-defined 1:1 orthologs (Sonnhammer and Östlund, 2015). Of these 1:1 orthologs, 667 are essential in fission yeast and can be tested for functional replaceability in assays that use growth as a readout (Kim et al., 2010), similar to the budding yeast assays we discussed above (Fig. 6C, Fig. 3). Fission yeast also better resemble humans in many cellular pathways, such as RNA interference, the splicing machinery, symmetric cell division, DNA replication mechanisms, and chromatin and telomere structure (Vo et al., 2016). Notably, many of these critical systems are non-replaceable in budding yeast (Kachroo et al., 2015). Furthermore, if genetic modularity is a universal principle driving functional complementation across species, many more distinct genetic modules could be humanizable in fission yeast compared to the budding yeast (as in Fig. 5C). For example, human PCNA is not replaceable in budding yeast but is in fission yeast, suggesting that the DNA replication machinery could be broadly replaceable in fission yeast (Waseem et al., 1992). This would allow researchers to use humanized fission yeast as a much-needed tractable model for the study of human DNA replication.

Although budding yeast provide significant experimental advantages to systematically test human gene function, the first successful humanization assays were actually performed in fission yeast (Lee and Nurse, 1987). Only a handful of fission yeast humanization experiments have been performed since the pioneering ones; nevertheless, the assays demonstrate the strength of fission yeast (Heinicke et al., 2007).

## Conclusions

In this Review, we highlight how, besides the obvious translational applications of humanized yeast, systematic replaceability studies provide fundamental insights into human gene function and evolution. Systematic functional replaceability experiments in yeast directly measure whether or not human and yeast genes are still functionally equivalent after a billion years of independent evolution. These assays therefore reveal features driving functional replaceability among highly diverged organisms. Finally, the systematic humanization assays further advance the already high relevance of distant model organisms for understanding human biology and disease.

Humanized yeast enables decoding human genetic variation at scale, providing a direct experimental measurement of the variant human gene activity. In addition, for most of the VUS, these will likely be the best predictors of clinical outcomes. The humanized yeast model could be explored to directly measure gene–drug interactions for any human allele–drug combinations, illuminating the principles of genetic disease with the potential to discover new therapeutics.

**Fig. 7.**
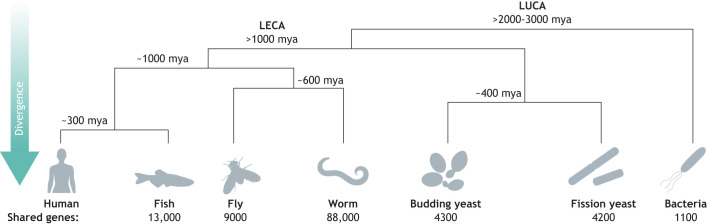
**Diverse humanized models beyond the yeast.** Despite millions of years of separate evolution from the last eukaryotic common ancestor (LECA) and last universal common ancestor (LUCA), humans share several thousand orthologs with many organisms, including fish, flies, worms and prokaryotes [data obtained from InParanoid (Sonnhammer and Östlund, 2015)]. In the future, scientists can explore humanizations in these diverse model organisms, which diverged several million years ago (mya), to directly measure human gene function.

Notably, systematic humanization assays can be performed in organisms other than budding yeast. Owing to common ancestry, every model organism in use today shares genes with humans. The less diverged an organism is, the more genes they share (Fig. 7). Although each model organism provides a unique framework and context to test functional conservation, implementation at scale could be a challenge. Nonetheless, humanization assays in higher eukaryotic models have been reported and serve as an outline for scalability (Balasov et al., 2020; Bellen et al., 2019; Rajan et al., 2020). However, none of these can yet match the throughput and tractability of budding yeast. Therefore, this simple eukaryote remains an invaluable asset in studying human gene function and the effect of human genetic variation on health, disease and evolution.

## Supplementary Material

Supplementary information
