# Supplementary material for: Humanized yeast to model human biology, disease and evolution
Source: Dis Model Mech. 2022 Jun 6;15(6):dmm049309. doi: 10.1242/dmm.049309 (PMC9194483; doi:10.1242/dmm.049309)
Supplement: Supplementary information [file dmm-15-049309-s1.pdf]

Table S1. List of all functionally replaceable human genes in yeast

Click here to download Table S1

Table S2. List of functionally replaceable human genes with associated OMIM or Orphanet disease phenotypes

Click here to download Table S2

Disease Models & Mechanisms • Supplementary information
